# Supplementary material for: Analysis of the Peripapillary and Macular Regions Using OCT Angiography in Patients with Schizophrenia and Bipolar Disorder
Source: J Clin Med. 2021 Sep 13;10(18):4131. doi: 10.3390/jcm10184131 (PMC8472507; doi:10.3390/jcm10184131)
Supplement: Supplementary file 1 [file jcm-10-04131-s001.zip › jcm-1345022-supplementary.pdf]

STATISTICAL ANALYSIS

The obtained results were analyzed statistically. The values of the analyzed measurable parameters were presented by means of the mean value, median and standard deviation, and for non-measurable ones—by the number and percentage. For measurable features, the normal distribution of the analyzed parameters was assessed using the Shapiro-Wilk test.
The Kruskal-Wallis test was used to compare multiple independent groups with the use of multiple comparison analysis. A significance level of $p < 0.05$ was adopted, indicating the existence of statistically significant differences. The database and statistical research were carried out on the basis of the STATISTICA 13.0 computer software (StatSoft, Poland).

Findings

The study included 70 patients (eyes), including 34.29% (n = 24) with schizophrenia, 22.86% (n = 16) with bipolar disorder and 42.85% (n = 30) from the control group.

The conducted studies did not show statistically significant differences in the assessment of individual RPC Density% parameters between the groups ($p > 0.05$), with the exception of the Whole capillary assessment ($p = 0.02$), where the values were significantly lower in the group with schizophrenia compared to the respondents with bipolar disorder or control. (table 1).

Supplementary Table S1. Evaluation of RPC Density% parameters in groups.

RPC Density %	Schizophrenia			Bipolar Disorder			Control Group			Statistical Analysis
	Mean	Median	SD	Mean	Median	SD	Mean	Mediana	SD	
Whole capillary	48.09	49.20	3.09	49.99	50.65	1.95	50.10	50.50	2.63	H=7.86, p=0.02*
superior	51.71	52.00	6.02	51.44	51.00	5.99	52.90	53.00	3.38	H=0.75, p=0.69
nasal	52.58	52.00	7.76	52.56	51.50	5.73	53.77	53.50	5.30	H=0.78, p=0.68
inferior	52.42	51.50	5.14	52.06	52.00	4.55	54.10	54.00	3.71	H=3.77, p=0.15
temporal	50.58	51.50	6.82	50.50	50.50	5.79	52.17	53.00	4.34	H=0.97, p=0.61

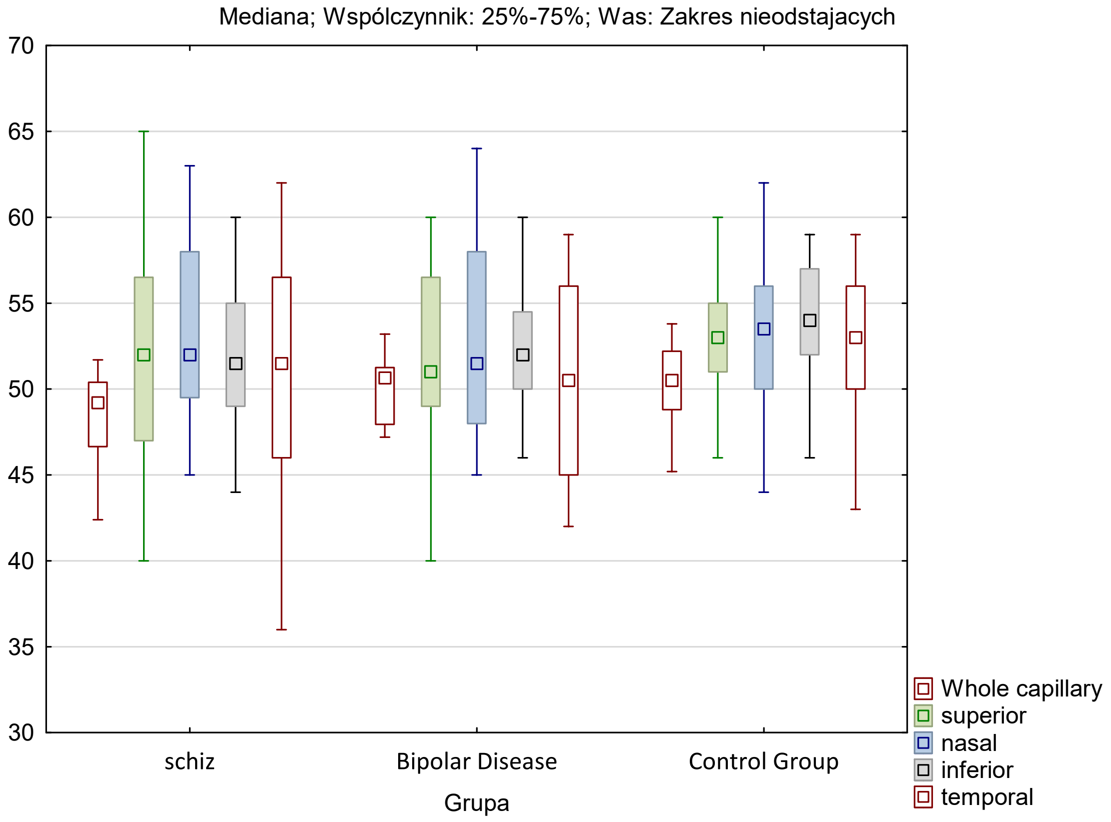

Supplementary Figure S1. Evaluation of RPC Density% parameters in groups

The conducted studies did not show statistically significant differences in the assessment of individual RPC RNFL μm parameters between the groups ($p > 0.05$) (Table 2).

Supplementary Table S2. Evaluation of RNFL μm parameters in groups

RNFL μm	Schizophrenia			BD			Control Group			Statistical Analysis
	Mean	Median	SD	Mean	Median	SD	Mean	Median	SD	
Peripapillary	118.09	114.00	23.56	113.07	114.00	20.45	112.57	116.00	9.01	H=0.98, p=0.61
Superior2	137.17	138.00	20.10	140.00	138.00	28.05	132.63	135.00	10.06	H=2.08, p=0.35
Nasal2	97.42	98.50	18.72	96.69	96.00	15.72	100.14	99.00	11.47	H=0.70, p=0.71
Inferior2	142.63	140.50	20.78	138.31	132.00	29.41	140.20	146.00	13.15	H=0.57, p=0.75
Temporal2	79.04	76.50	12.84	76.69	68.50	16.78	76.60	79.00	11.16	H=0.90, p=0.64

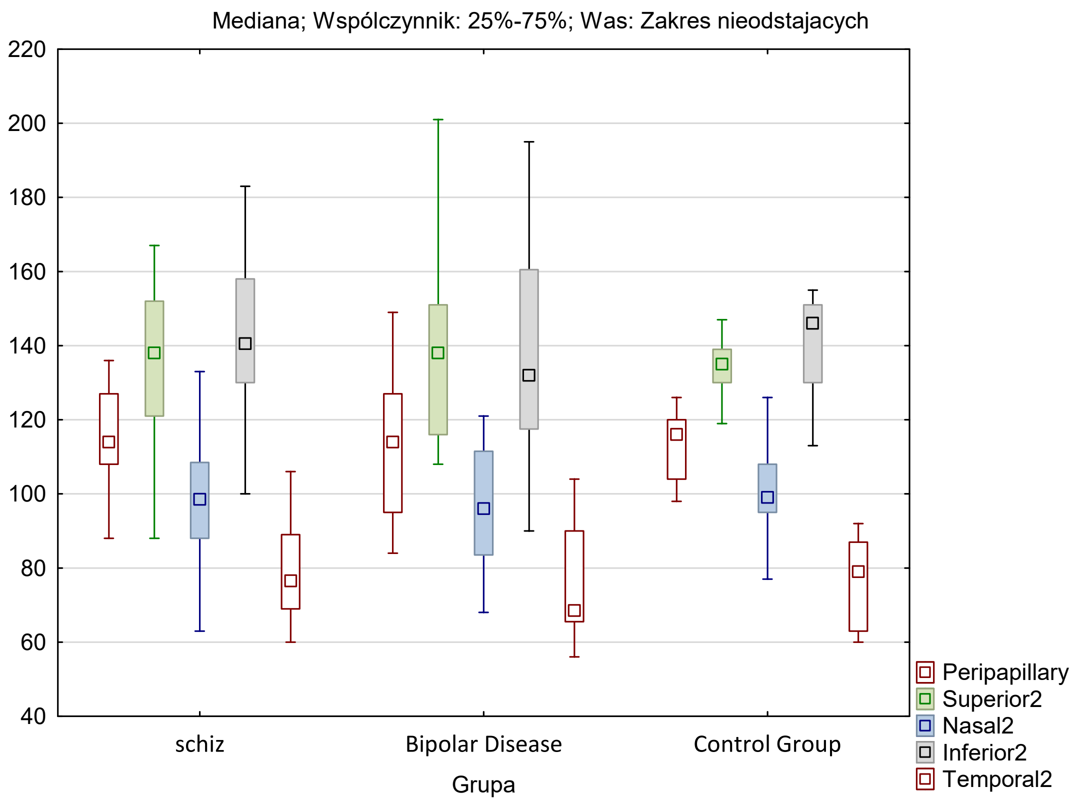

Supplementary Figure S2. Assessment of RNFL μm parameters in groups.

Statistical analysis showed highly significant differences between the groups as assessed by Whole deep ($p < 0.0001$), where values were significantly lower in the group with schizophrenia. The analysis of multiple comparisons showed differences between the group with schizophrenia and the control group ($Z = 4.30$; $p = 0.00005$) and between the control group and the group with bipolar disorder ($Z = 3.09$; $p = 0.006$). control and bipolar group ($p > 0.05$). However, no significant differences were found between the groups in the assessment of the remaining parameters of Macula/Density% ($p > 0.05$) (Table 3).

Supplementary Table S3. Evaluation of Macula / Density% parameters in groups

**Macula/Density %**	**Schizophrenia**			**BD**			**Control Group**			**Statistical Analysis**
	**Mean**	**Median**	**SD**	**Mean**	**Median**	**SD**	**Mean**	**Median**	**SD**	
Whole sup.	47.93	48.40	3.37	48.56	48.60	3.13	47.80	48.10	2.84	H=0.55, p=0.76
Whole deep	43.66	42.45	5.50	50.41	49.25	6.54	50.66	51.45	4.26	H=19.91, p<0.0001*
Fovea sup.	24.15	24.95	6.96	23.59	21.45	9.45	21.27	20.75	3.86	H=3.00, p=0.22
Fovea deep	38.81	40.40	7.74	39.38	37.40	8.64	38.43	39.00	4.87	H=1.15, p=0.56

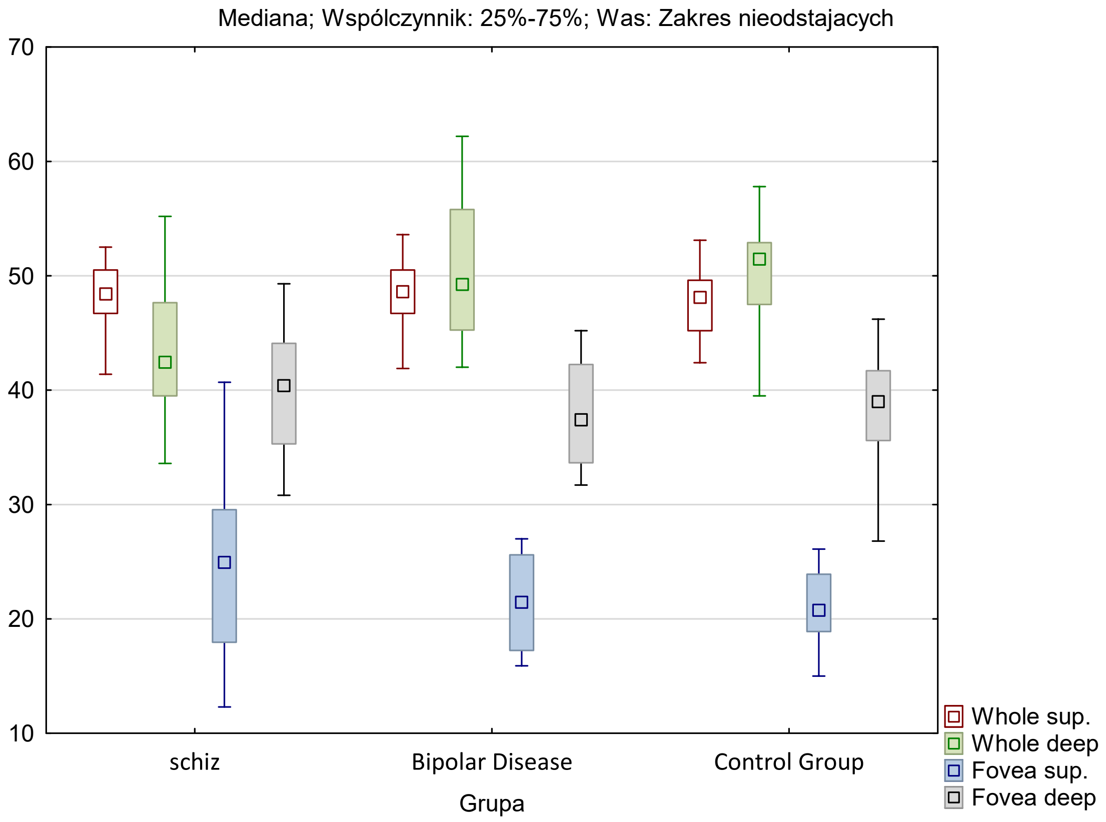

Supplementary Figure S3. Assessment of Macula/Density% parameters in groups.

Statistical analysis showed highly significant differences between the groups in terms of Whole ($p < 0.0001$) and Fovea ($p = 0.02$). The analysis of multiple comparisons showed that the Whole parameter was significantly higher in the control group compared to the group with schizophrenia ($Z = 4.53$; $p = 0.00002$) and compared to the group with bipolar disorder ($Z = 5.03$; $p = 0.000001$), however, no differences in the Whoole assessment were found between schizophrenia and bipolar disorder ($p > 0.05$). In the evaluation of the Fovea parameter, in the analysis of multiple comparisons, differences were found between the control group and bipolar disorder ($Z = 2.71$; $p = 0.02$), while between the other groups the differences were not statistically significant ($p > 0.05$).

Supplementary Table S4. Evaluation of Macula thickness µm parameters in groups

Macula Thickness µm	Schizophrenia			BD			Control Group			Statistical Analysis
	Mean	Median	SD	Mean	Median	SD	Mean	Median	SD	
Whole	284.25	283.00	14.77	278.50	273.50	12.59	305.23	301.50	11.33	H=33.05, p<0.0001*
Fovea	260.67	262.00	24.28	249.63	244.00	21.43	263.53	263.50	15.37	H=7.41, p=0.02*

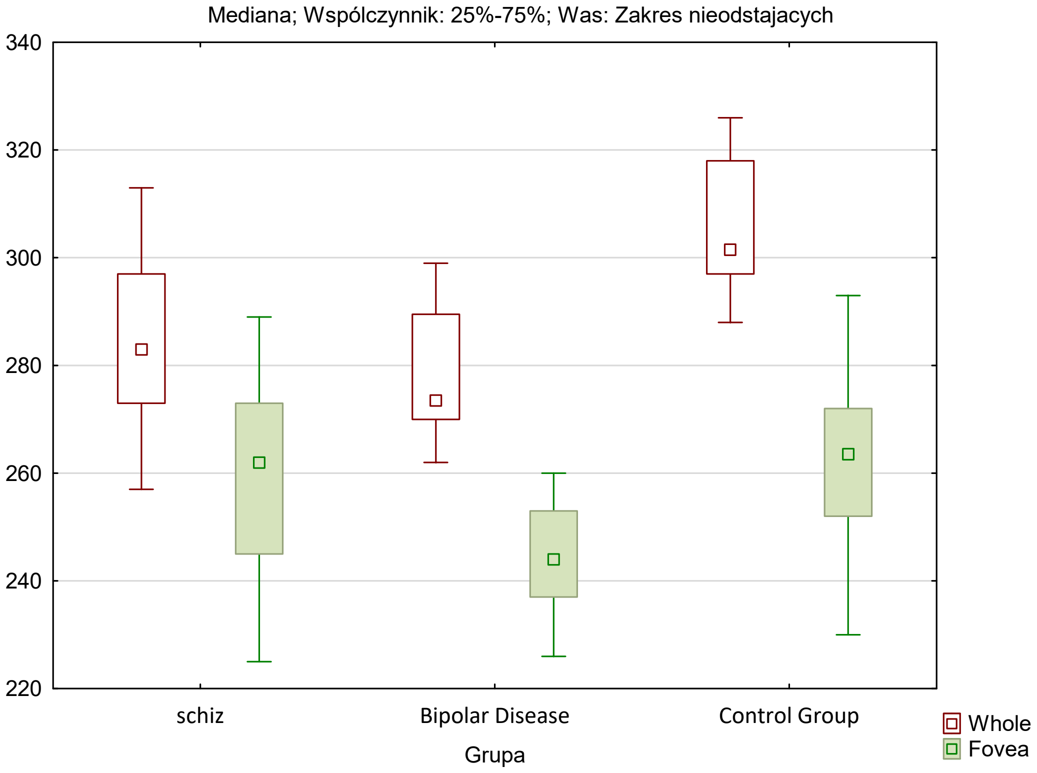

Supplementary Figure S4. Assessment of Macula thickness μm parameters in groups.
